# Butterflies in urban parks in the Bangkok Metropolitan Region, Thailand

**DOI:** 10.3897/BDJ.8.e56317

**Published:** 2020-10-12

**Authors:** Narong Jaturas, Kong-Wah Sing, John-James Wilson, Hui Dong

**Affiliations:** 1 Department of Microbiology and Parasitology, Faculty of Medical Science, Naresuan University, Phitsanulok, Thailand Department of Microbiology and Parasitology, Faculty of Medical Science, Naresuan University Phitsanulok Thailand; 2 Arthropod Ecology and Biological Control Research Group, Ton Duc Thang University, Ho Chi Minh City, Vietnam Arthropod Ecology and Biological Control Research Group, Ton Duc Thang University Ho Chi Minh City Vietnam; 3 Faculty of Applied Sciences, Ton Duc Thang University, Ho Chi Minh City, Vietnam Faculty of Applied Sciences, Ton Duc Thang University Ho Chi Minh City Vietnam; 4 Vertebrate Zoology at World Museum, National Museums Liverpool, Liverpool, United Kingdom Vertebrate Zoology at World Museum, National Museums Liverpool Liverpool United Kingdom; 5 Fairy Lake Botanical Garden, Shenzhen & Chinese Academy of Sciences, Shenzhen, China Fairy Lake Botanical Garden, Shenzhen & Chinese Academy of Sciences Shenzhen China

**Keywords:** Bangkok, butterflies, DNA barcodes, parks, Southeast Asia, Thailand, urban

## Abstract

**Background:**

For residents of East-Southeast Asia’s megacities, interactions with “nature” may be largely limited to interactions taking place in urban parks. Urban parks provide refuges for ecologically-important biodiversity, such as insect pollinators. While residents may be unlikely to notice small insects, butterflies are more likely to be noticed and to provide positive human-“nature” interactions. Engaging residents and city planners in promoting habitat for butterflies is valid conservation practice and has well-understood educational and well-being benefits. Surveying and monitoring is an essential activity to corroborate, improve and communicate the outcomes of conservation practices amongst city governments, scientists and other stakeholders. Here we present the data from a survey of butterflies in urban parks in the megacity of the Bangkok Metropolitan Region as part of the "Urban biodiversity and human well-being in East-Southeast Asia's megacities" project organised by the "Urban Butterflies in Asia Research Network".

**New information:**

We recorded 51 species of butterflies from ten urban parks in the Bangkok Metropolitan Region. This was more than double the 25 species reported in Bangkok's City Biodiversity Index application. However, this was lower than that recorded in other megacities in Southeast Asia, such as Kuala Lumpur at 60 species. Most of the butterflies recorded were common and widespread species. DNA barcodes are provided for most of the butterflies sampled.

## Introduction

East-Southeast Asia has seen the fastest rates of urbanisation globally (Schneider et al. 2015). A consequence of urbanisation is that residents have less exposure to “nature”. The loss of an emotional connection with nature is closely associated with not only the decline in people’s willingness to protect nature (Imai et al. 2018), but also reduced psychological well-being (Soga and Gaston 2016). Urban planning often incorporates public parks, providing improved air quality and opportunities for recreation. For residents of East-Southeast Asia’s megacities, interactions with “nature” may be largely limited to interactions taking place in urban parks. While residents may be unlikely to notice small insects such as bees, butterflies are more likely to be noticed and to provide positive human-“nature” interactions (Wilson et al. 2015). Engaging residents and city planners in promoting habitat for butterflies is valid conservation practice and has well-understood educational and well-being benefits (Hall et al. 2017). Surveying and monitoring is an essential activity to corroborate, improve and communicate the outcomes of conservation practices amongst city governments, scientists and other stakeholders (Hall et al. 2017, Ramírez-Restrepo and MacGregor-Fors 2016). This is clearly demonstrated by *change in number of butterfly species* being a mandatory indicator in the City Biodiversity Index (or Singapore Index) (Secretariat Convention on Biological Diversity 2013, Uchiyama and Kohsaka 2019). Here we present the data from a survey of butterflies in urban parks in the megacity of the Bangkok Metropolitan Region as part of the "Urban biodiversity and human well-being in East-Southeast Asia's megacities" project organised by the "Urban Butterflies in Asia Research Network" (Sing et al. 2017, Wilson and Sing 2020). Twenty-five species of butterflies have previously been reported within the city limits of Bangkok according to the City Biodiversity Index application (dated October 2012), but in view of the species richness of the region, this seems an underestimate. We recorded 51 species of butterflies from ten urban parks in the Bangkok Metropolitan Region.

## Project description

### Title

Urban biodiversity and human well-being in East-Southeast Asia’s megacities

### Personnel

The "Urban Butterflies in Asia Research Network" involves researchers across East-Southeast Asia. Information can be found on the project's website (Wilson and Sing 2020) and *ResearchGate* project page (https://www.researchgate.net/project/Urban-Butterflies-in-Asia-Research-Network).

### Study area description

**Figure 1.**
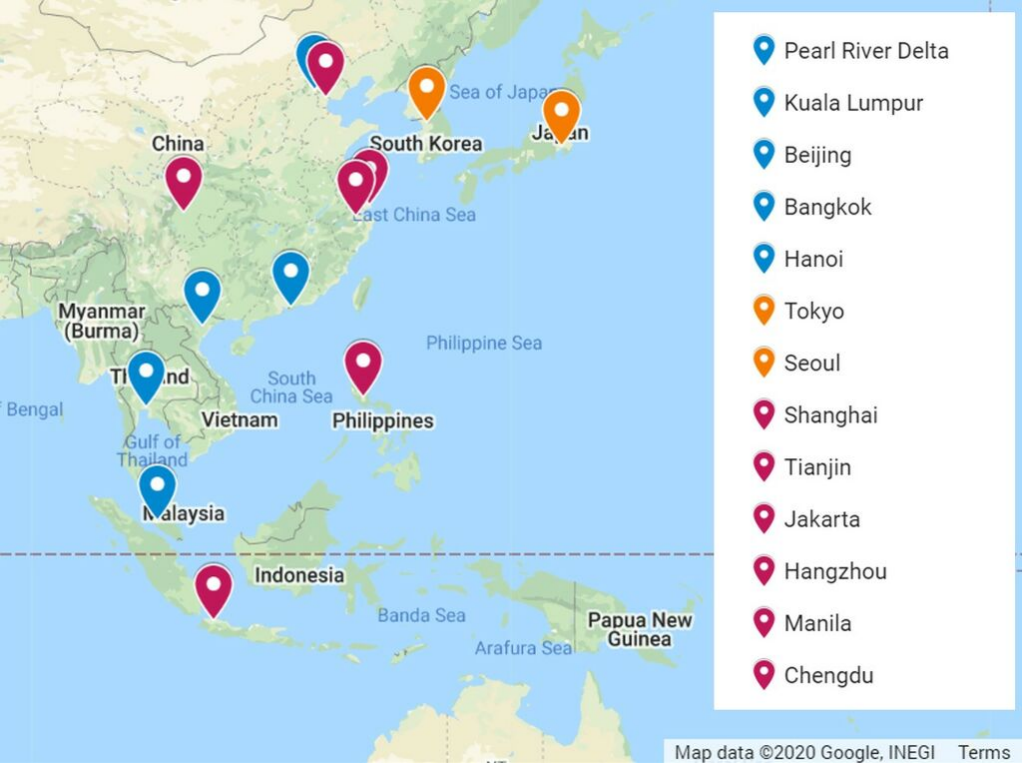
Megacities in East-Southeast Asia featured in Figure 1 of Schneider et al. (2015). Butterfly sampling has been completed in urban parks by the "Urban Butterflies in Asia Research Network" (Sing et al. 2017, Sing et al. 2016a, Sing et al. 2016b, Sing et al. 2019, Wilson and Sing 2020) at those megacities with blue markers. Butterfly sampling has been completed in urban parks by other researchers (Matsumoto 2015, Nagase et al. 2019, Lee et al. 2015) at those megacities with orange markers. To our knowledge, no butterfly sampling has been published for urban parks at those megacities with magenta markers.

In urban green spaces, such as city parks, native insects provide important ecosystem services including pollination of plants that provide food for humans and other animals and enrich human well-being. These important services proceed largely unnoticed and have received limited attention. Several studies of insect diversity in city parks, thought of as urban wildlife refuges, have been conducted in Europe and North America, but few have been conducted in rapidly urbanising countries in East-Southeast Asia (Ramírez-Restrepo and MacGregor-Fors 2016). Without further research into the diversity of insects in urbanisation hotspots, we cannot predict how future development will affect the ecosystem services and benefits they provide. Our project focuses on megacities across the region (Fig. 1) and on butterflies – a model “biodiversity indicator” group for biodiversity studies (Syaripuddin et al. 2015).

### Design description

We will (1) generate data from urban parks in megacities across East-Southeast Asia to enable region-wide meta-analyses of butterfly diversity in this rapidly urbanising region (Sing et al. 2017); (2) examine the value of urban parks as refuges for butterflies through investigating the relationships between butterfly species richness and the age, size and distance from the central business district of parks in East-Southeast Asian cities (Sing et al. 2016a, Sing et al. 2016b, Sing et al. 2019); (3) identify which type of microhabitat within urban parks provides suitable breeding and foraging habitat for butterflies (Sing et al. 2016a, Sing et al. 2016b); and (4) contribute to DNA barcode reference libraries of urban butterflies to enable rapid surveys of these species in future studies (Wilson et al. 2013).

### Funding

The project has received funding from the Asia-Pacific Network for Global Change Research (CRYS2017-03SY-Sing); Fundamental Research Funds for the Central Nonprofit Research Institution of CAF (CAFYBB2020ZB008); Biodiversity Conservation Programme of the Ministry of Ecology and Environment, China (China-BON Butterflies) (SDZXWJZ01059-2018). We have also received support from the Kunming Institute of Zoology, Chinese Academy of Sciences; Fairy Lake Botanical Garden, Shenzhen & Chinese Academy of Sciences; Naresuan University, Thailand. We are grateful to all the park managers and local authorities who have provided permission to conduct butterfly surveys and supported the project (in kind) in many ways.

## Sampling methods

### Study extent

**Figure 2.**
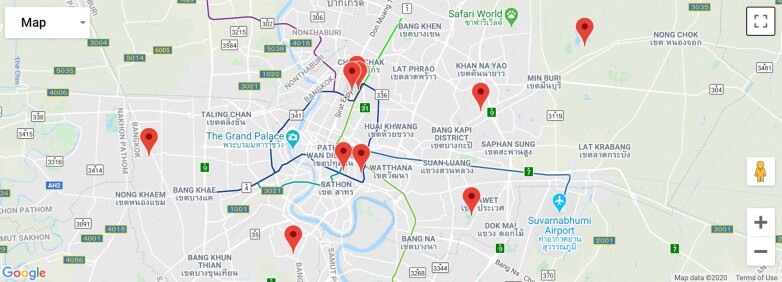
Location of ten parks within the Bangkok Metropolitan Region where we conducted butterfly surveys.

Ten parks managed by the Bangkok Metropolitan Authority were selected for sampling (Fig. 2). These represent a range of park areas, ages and degree of urbanisation i.e. distance from the urban core.

### Sampling description

Each park was sampled over three consecutive days comprising 180 minutes each day. We followed an active search-timed survey method used in our butterfly surveys in Kuala Lumpur (Sing et al. 2016b), Shenzhen (Sing et al. 2016a) and Beijing (Sing et al. 2019), where butterflies were collected during the 180 minutes survey period within accessible areas. This method allowed a full search of green areas in parks and avoided sampling biases due to differences in size and shape between parks. Butterflies were collected by an experienced collector using a hand net between 09:00 h and 14:00 h during calm weather to correspond with the peak flight activity period of most adult butterflies.

### Quality control

**Sampling limitations**

While we followed a standardised sampling approach to aid comparison with surveys conducted in other megacities, the number of species recorded will have been limited by the collecting method (i.e. hand net only, no bait trapping), time of day (i.e. crepusular species could be missed) and the season (i.e. some species will not have been present as adults at this time of year).

**Species identification**

We combined both morphological methods (i.e. comparision with images and descriptions in Ek-Amnuay 2012) and DNA barcoding (i.e. matches in the DNA barcode library) for species identification. All collected butterflies were brought back to the university. DNA was extracted from a single leg, or 2–3 legs in the case of small lycaenids, of each butterfly using the TIANamp extraction kit following the manufacturer’s instructions (Tiangen Biotech, Beijing). DNA barcode fragments of COI mtDNA were amplified (following standard protocols in Wilson 2012) using LCO1490/HCO2198 primers. PCR products were Sanger-sequenced and checked for quality (following standard protocols in Wilson et al. 2019). Specimen data and any generated DNA barcodes were submitted to Barcode of Life Datasystems (BOLD; Ratnasingham and Hebert 2007). In BOLD, the DNA barcodes were automatically sorted into Barcode Index Numbers (BINs; Ratnasingham and Hebert 2013) which greatly aided the application of names to specimens with sequences. At present, five species could only be given a genus name (from genera, *Borbo*, *Parnara* and *Pelopidas*) (Suppl. material 1); however, these may be updated as the BOLD library grows and the taxonomy applied in BOLD is refined.

## Geographic coverage

### Description

Bangkok Metropolitan Region, Thailand

### Coordinates

13.495 and 13.955 Latitude; 100.328 and 100.938 Longitude.

## Taxonomic coverage

### Taxa included

**Table:** 

Rank	Scientific Name	Common Name
superfamily	Papilionoidea	True butterflies
superfamily	Hesperioidea	Skipper butterflies

## Temporal coverage

**Data range:** 2017-7-30 – 2017-8-18.

## Usage rights

### Use license

Creative Commons Public Domain Waiver (CC-Zero)

## Data resources

### Data package title

Bangkok Urban Butterflies

### Resource link

http://www.boldsystems.org/index.php/Public_SearchTerms?query=DS-BKKUB

### Number of data sets

2

### Data set 1.

#### Data set name

DS-BKKUB (Bangkok Urban Butterflies)

#### Data format

DWC; XML; TSV

#### Number of columns

26

#### Download URL

dx.doi.org/10.5883/DS-BKKUB

#### Description

The dataset contain 693 records and 438 COI DNA barcodes grouped into 43 BINS. Below are listed the columns for the DWC format.

**Data set 1.** 

Column label	Column description
id	BOLD Process ID
occurrenceID	BOLD Process ID
catalogNumber	BOLD Specimen ID
fieldNumber	BOLD Specimen ID/Field number
identificationRemarks	BIN number
basisOfRecord	Because these records are held on the Barcode of Life Datasystems this is recorded as "DNA Barcode"
institutionCode	Code for the institution currently holding the physical specimen
phylum	Taxonomic phylum
class	Taxonomic class
order	Taxonomic order
family	Taxonomic family
genus	Taxonomic genus
scientificName	Taxonomic species name
identifiedBy	Taxonomic determiner
habitat	Collection habitat
eventDate	Collection date
recordedBy	Collector
country	Collection country
stateProvince	Collection state/province
locality	Precise collection locality/urban park
decimalLatitude	Decimal latitude
decimalLongitude	Decimal longitude
lifestage	Lifestage of the specimen when collected
rightsHolder	Holder of the rights
rights	Creative commons licence applied
language	Language used for the record

### Data set 2.

#### Data set name

Bangkok Urban Parks Butterfly Species Checklist

#### Data format

Excel 97-2003 Workbook (*.xls)

#### Number of columns

13

#### Download URL

see Supplementary files

#### Description

A checklist of species found in each urban park (Suppl. material 1).

**Data set 2.** 

Column label	Column description
Butterfly family	Butterfly family
Butterfly species	Butterfly species
Barcode Index Number (BIN) for the species	Barcode Index Number (BIN) for the species [Inferred BIN in the case where specimens were not sequenced is given in parentheses]
Benchakitti Park	Species recorded in Benchakitti Park (indicated by a "Y")
Chatuchak Park	Species recorded in Chatuchak Park (indicated by a "Y")
Lumphini Park	Species recorded in Lumphini Park (indicated by a "Y")
Queen Sirikit Park	Species recorded in Queen Sirikit Park (indicated by a "Y")
Rama IX Park	Species recorded in Rama IX Park (indicated by a "Y")
Seri Thai Park	Species recorded in Seri Thai Park (indicated by a "Y")
Thonburirom Park	Species recorded in Thomburirom Park (indicated by a "Y")
Thawee Wanarom Park	Species recorded in Thawee Wanarom Park (indicated by a "Y")
Wachirabenchatat Park	Species recorded in Wachirabenchatat Park (indicated by a "Y")
Wareepirom Park	Species recorded in Wareepirom Park (indicated by a "Y")

## Supplementary Material

8041BDFC-91B2-50DE-867F-0796D639A7CC 10.3897/BDJ.8.e56317.suppl1 Supplementary material 1 Bangkok Urban Parks Butterfly Species Checklist Data type Species occurences File: oo_446041.xls https://binary.pensoft.net/file/446041 Narong Jaturas, Kong-Wah Sing, John-James Wilson, Hui Dong
